# Natural Variation in *SlSPS1* Promoter Reduces Soluble Solids Content During Tomato Domestication

**DOI:** 10.1111/pbi.70751

**Published:** 2026-08-27

**Authors:** Yonglian Wang, Xin Li, Can Zhu, Saifeng Guo, Junling Hu, Yunhao Han, Shumin He, Yujie Zhang, Chen Zhang, Zejun Huang, Xiaoxuan Wang, Yanmei Guo, Yongchen Du, Lin Yang, Junming Li, Lei Liu

**Affiliations:** ^1^ State Key Laboratory of Vegetable Biobreeding, Institute of Vegetables and Flowers Chinese Academy of Agricultural Sciences Beijing China; ^2^ China Vegetable Seed Technology Co., Ltd. (Beijing) Beijing China

**Keywords:** domestication, promoter, *SlSPS1*, soluble solids content, tomato

Soluble solids content (SSC), an essential determinant of tomato flavour, nutrition and processing quality, is mainly composed of glucose and fructose derived from sucrose hydrolysis. This sucrose is synthesized from photosynthates in source organs, translocated to the fruit and cleaved into hexoses for vacuolar accumulation (Ruan et al. 1997). In tomato, numerous quantitative trait loci (QTLs) and causal genes underlying high SSC have been mapped to downstream sucrose metabolism, including genes involved in sucrose cleavage, sugar transport, and storage, such as *LIN5*, *STP1*, *SWEETs* and *CDPK27* (Fridman et al. 2002; Ko et al. 2021; Wang et al. 2023; Zhang et al. 2024). Upstream regulators of sucrose biosynthesis have been identified in other crops: in apple, the sucrose phosphate synthase gene *MdSPS* is transcriptionally activated by *MdWRKY126* and promotes sucrose accumulation in fruit cells (Zhang et al. 2023, 2025). By contrast, the enzymatic steps governing de novo sucrose synthesis in tomato—particularly the natural allelic variants and regulatory mechanisms of SPS—remain poorly characterized. Elucidating these upstream components is therefore a critical frontier for rationally enhancing tomato fruit quality.

Wild tomato germplasm harbours elite alleles for high SSC that were lost during domestication. By evaluating over 300 accessions of *Solanum pimpinellifolium* , we identified LA1924, an accession notable for its stable high SSC across 3 years and three distinct environments. Fruit SSC in an F_2_ population (*n* = 300) derived from LA1924 × Moneymaker (MM) followed a near‐normal distribution (Figure S1), consistent with quantitative inheritance of this trait. Bulked segregant analysis sequencing (BSA‐seq) and SNP‐index mapping of high‐ and low‐SSC pools resolved three robust QTLs for fruit SSC (Figure 1a). Based on prior reports and our sequence alignments, *qSSC2.1* and *qSSC9.1* co‐localized with the known functional variants that underlie *STP1* and *LIN5*, respectively (Figures S2 and S3). Notably, *qSSC7.1* is a previously uncharacterized locus for elevated fruit SSC and was mapped to chromosome 7 between markers 7‐B12 and 7‐B15 (Figure 1b).

**FIGURE 1:**
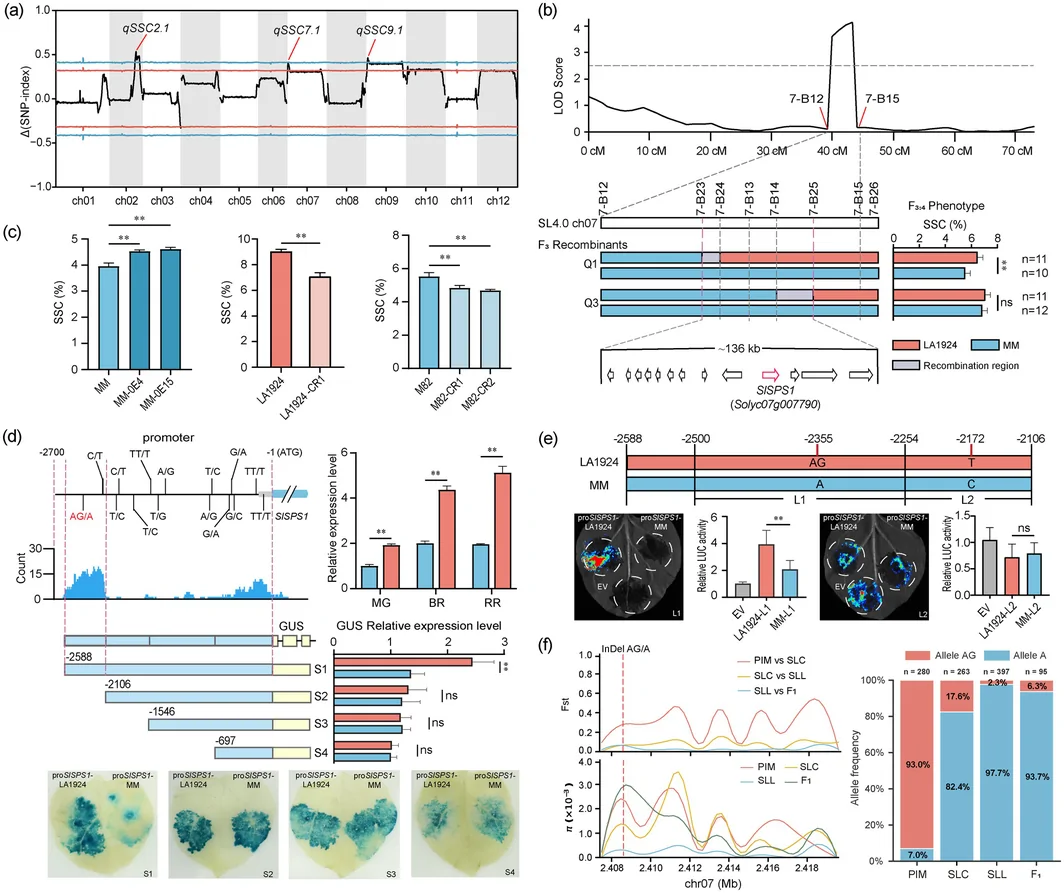
Identification of *SlSPS1* as a domesticated gene underlying tomato SSC. (a) BSA‐seq identified QTLs for tomato SSC. (b) Fine‐mapping of *qSSC7.1*. (c) Compared with the control, *SlSPS1*‐overexpressing lines and edited lines display significantly different fruit SSC. (d) Natural promoter variations, ATAC‐seq signals, truncated fragments drive differential β‐glucuronidase (GUS) activity and *SlSPS1* relative expression in MM (blue) and LA1924 (orange) during fruit developmental stages. S1 (−2588 to −1 bp), S2 (−2106 to −1 bp), S3 (−1546 to −1 bp), S4 (−697 to −1 bp). Only S1 exhibits significant allelic differences. MG (mature green), BR (breaker), RR (red ripe). (e) Dual‐luciferase assays identify the key functional variant. L1 (−2500 to −2265 bp), L2 (−2254 to −2107 bp). LA1924‐L1 confers significantly higher LUC activity relative to MM‐L1. (f) The AG/A indel in the *SlSPS1* promoter is under strong domestication selection. PIM ( *Solanum pimpinellifolium* ), SLC ( *Solanum lycopersicum* var. *cerasiforme* ), SLL (cultivated *S. lycopersicum* ) and F_1_ (F_1_ hybrids). ‘*’ *p* < 0.05, ‘**’ *p* < 0.01; ns, not significant.

For fine‐mapping of *qSSC7.1*, we expanded the population to 2100 F_2_ plants and screened for key recombinants. Three individuals carrying crossovers within *qSSC7.1*—while homozygous at *qSSC2.1* and *qSSC9.1*—were selected to eliminate genetic background interference. Genotyping and phenotyping of their F_2:3_ and F_3:4_ progenies narrowed *qSSC7.1* to a 135.38‐kb interval between markers 7‐B23 and 7‐B25 (Figure S4). Of the 13 annotated genes in this interval (Table S5), *SlSPS1* (*Solyc07g007790*) was prioritized as the top candidate based on its functional annotation, sequence variants, and expression profiles. *SlSPS1* encodes sucrose phosphate synthase, an enzyme that is essential for sugar accumulation. Although *SlSPS*1 has been implicated in growth and thermotolerance (Zhang et al. 2022), natural variants at this locus with a major impact on sugar accumulation have not been reported. Consistent with its potential role in sucrose biosynthesis, *SlSPS1* displayed fruit‐preferential expression across tissues (Figure S5), with a marked increase in transcript abundance during ripening in LA1924 compared to MM (Figure 1d). To functionally validate its role in fruit SSC, we generated 35S‐driven *SlSPS1* overexpression lines in MM background and CRISPR/Cas9 knockout lines in LA1924 and M82 backgrounds. Overexpression elevated fruit SSC, whereas knockout reduced it, with transcript levels showing a significant positive correlation with SSC; fruit weight was not significantly altered in any transgenic line (Figure 1c; Figure S6). These results demonstrate that *SlSPS1* is the causal gene underlying *qSSC7.1* and positively contributes to tomato fruit SSC.

To elucidate the basis for differential *SlSPS1* expression between LA1924 and MM, we analysed sequence variations across the gene. The coding region contained only two synonymous single nucleotide polymorphisms (SNPs), whereas the 2.7 kb promoter harboured 15 SNPs and insertions/deletions (InDels) (Figure 1d; Table S6). Integration of tomato fruit assay for transposase‐accessible chromatin sequencing (ATAC‐seq) data (Guo et al. 2023) revealed that the promoter has two regions of open chromatin (−2.14 to −2.62 kb and 0 to −0.44 kb) (Figure 1d), defining them as core regulatory modules. To pinpoint the causal variant among these promoter polymorphisms, we generated four serially 5′‐truncated promoter fragments, designated S1, S2, S3, and S4. Transient expression assays narrowed the differential activity between LA1924 and MM to a 483‐bp region (−2588 to −2106 bp of S1), an effect that was independently corroborated by dual‐luciferase assays (Figure 1d; Figure S7). Further truncation of the 483‐bp fragment localized the differential activity to fragment L1 and identified a 1‐bp InDel (AG/A at −2355 bp) as the causal variant underlying the divergent *SlSPS1* promoter activity (Figure 1e). The promoter InDel not only accounts for phenotypic variation in SSC but also provides a critical entry point for dissecting the transcriptional control of *SPS*. Consistent with its functional importance, this 1‐bp InDel displays clear signatures of selection, as evidenced by analysis of 1035 resequenced accessions across four tomato domestication and improvement stages: the high‐SSC AG allele declined markedly during early domestication, then partially recovered in contemporary quality‐focused programs (Figure 1f). This trajectory identifies the InDel as a promising breeding target for enhancing fruit SSC and quality via the sucrose synthesis pathway.

Breeding tomato for high and stable SSC remains a major challenge. The 1‐bp InDel in the *SlSPS1* promoter fine‐tunes SPS expression and underlies natural SSC variation. Distinct from single‐gene manipulation, we propose that combining modulation of SPS with other sugar metabolic genes could confer metabolic homeostasis and sustain elite SSC performance.

## Author Contributions

Y.W. and X.L. performed the experiments, analysed the data, and wrote the manuscript; C.Z., S.G., J.H., Y.H., S.H., and Y.Z. helped perform data collection and analysis; Z.H., X.W., Y.G., Y.D. and L.Y. revised the manuscript; L.L. and J.L. designed the experiments, and revised the manuscript. All authors confirmed the final manuscript.

## Funding

This research was supported by the Corps Science and Technology Plan Project (2024AB022), the Science and Technology Innovation Program of the Chinese Academy of Agricultural Sciences (CAAS‐ASTIP‐IVFCAAS), and the Key Laboratory of Biology and Genetic Improvement of Horticultural Crops (Vegetables), Ministry of Agriculture and Rural Affairs, P.R. China.

## Conflicts of Interest

The authors declare no conflicts of interest.

## Supporting information

comprising Figures S1‐S7 and Tables S1‐S7.

## Data Availability

The primer sequences have been provided within the Supporting Information files. The raw BSA‐seq were deposited in the NCBI Sequence Read Archive under the BioProject accession number PRJNA1461373.
