# Supplementary material for: Natural Variation in SlSPS1 Promoter Reduces Soluble Solids Content During Tomato Domestication
Source: Plant Biotechnol J. 2026 Aug 27;24(11):5845–7. doi: 10.1111/pbi.70751 (PMC13519583; doi:10.1111/pbi.70751)
Supplement: Supplementary file 1 [file PBI-24-5845-s001.docx]

**Supporting information**

**Natural variation in *SlSPS1* promoter reduces** **soluble solids content during tomato domestication**

Yonglian Wang^1,†^, Xin Li^1,†^, Can Zhu^1,†^, Saifeng Guo^1^, Junling Hu^1^, Yunhao Han^1^, Shumin He^1^, Yujie Zhang^1^, Chen Zhang^1^, Zejun Huang^1^, Xiaoxuan Wang^1^, Yanmei Guo^1^, Yongchen Du^1^, Lin Yang^1,2^, Junming Li^1,^*, Lei Liu^1,^*

^1^State Key Laboratory of Vegetable Biobreeding, Institute of Vegetables and Flowers, Chinese Academy of Agricultural Sciences, Beijing, China

^2^ China Vegetable Seed Technology Co., Ltd. (Beijing), Beijing 100081, China

**Figure S1. SSC phenotypes in parental lines, F_1_, and F_2_ progeny.** (a) Phenotypic comparison of SSC in Moneymaker (MM), LA1924 and F_1_. Data are shown as mean ± SD. Significant differences were analyzed by Student’s *t-test*; ** indicates *P* < 0.01. (b) Frequency distribution of SSC in the F_2_ population (n=300). The dashed line represent the fitted normal distribution curve.

**Figure S2. Linkage mapping of *qSSC2.1* and functional variation in candidate gene *STP1*.** (a) Linkage mapping of *qSSC2.1* on chromosome 2. The genetic distances (cM) and molecular markers (2-B1 to 2-B14) of the interval are shown on the left, while the LOD (logarithm of the odds) scores for the trait are plotted on the right. The LOD curve identifies the most likely genomic region underlying the quantitative trait. (b) Identification of a 21-bp insertion/deletion (InDel) in the *STP1* promoter. The top panel shows the reference genome sequence (SL4.0) of the *STP1* promoter region with the relative positions (−1125 and −1124 bp) indicated. The middle and bottom panels display the Sanger sequencing chromatograms and sequence alignments for the LA1924 and MM genotypes, respectively. A 21-bp insertion (outlined in the red box) was specifically identified in the promoter region of LA1924, whereas MM and the reference sequence lack this InDel, highlighting a potential functional variation within the mapped candidate region.

**Figure S3. Linkage mapping of *qSSC9.1* and sequence analysis of functional variations in *LIN5*.** (a) Linkage map and LOD score plot for *qSSC9.1* on chromosome 9. The molecular markers (9-B1 to 9-B13) and the corresponding LOD curve delineate the primary mapping interval, with the peak LOD score approaching 10. (b) Identification of a single nucleotide polymorphism (SNP) within the *LIN5* coding region. The amino acid sequence alignment for the reference genome (SL4.0) is shown at the top. The blue box indicates a previously reported variation in *LIN5* (the N/D amino acid variation in the SL4.0 protein sequence). The red box highlights the specific polymorphic SNP site that distinguishes LA1924 from the wild-type allele (MM) and the SL4.0 reference. The corresponding Sanger sequencing chromatograms for LA1924 and MM are shown beneath the sequence alignment, confirming the presence of this variation.

**Figure S4. Mapping of *qSSC7.1* in key F_2:3_ recombinant families and fruit SSC phenotype.** Genotypes and corresponding fruit SSC phenotypes of F2:3 recombinant families (C1–C3). Horizontal bars indicate the marker genotypes within the qSSC7.1 interval for each recombinant family, with red representing the LA1924 allele, blue representing the Moneymaker allele, and gray indicating the recombination region. The bar chart on the right shows the mean ± SD SSC of fruits for each genotype. Significant differences were assessed by Student’s t-test; ** indicates P < 0.01 and ns indicates no significant difference.

**Figure S5.** Expression pattern of *SlSPS1* across different tomato tissues and fruit developmental stages (Waese et al., 2017; https://bar.utoronto.ca/efp_tomato/cgi-bin/efpWeb.cgi). Color intensity from yellow to red indicates increasing transcript abundance.

**Figure S6. Characterization of *SlSPS1* CRISPR/Cas9-edited and overexpression lines.** (a) The gene structure of *SlSPS1* and CRISPR/Cas9 target sites. Sequence alignments of the target regions in the wild type (WT) and edited lines are shown below. (b) Relative transcript levels of *SlSPS1* in WT, overexpression (OE), and gene-edited lines. The OE and CRISPR/Cas9-edited lines display significantly different expression levels compared to WT. (c) Comparison of fruit weight among WT, OE, and edited lines. No significant difference in fruit weight was observed between the overexpression and gene-edited lines when compared to WT. Data are presented as mean ± SD. Significant difference was determined by Student’s *t*-test; ** indicates *P* < 0.01, and ns indicates no significant difference.

**Figure S7. Validation of the key regulatory fragment of the *SlSPS1* promoter using dual-luciferase transient expression assays.** (a) The S1 fragment from LA1924 showed significantly higher promoter activity than that from MM. (b) No significant difference in promoter activity was detected between the S2 fragments derived from the two parental lines. Data are presented as mean ± SD. Significant difference was determined by Student’s t-test; * indicates *P* < 0.05 and ns indicates no significant difference.

**Table S1 Sequencing quality and read mapping statistics for BSA-seq samples**.

| **Sample** | **Clean bases (G)** | **Q20 (%)** | **Q30 (%)** | **GC (%)** | **Mapped reads (%)** | **Coverage (%)** | |
| --- | --- | --- | --- | --- | --- | --- | --- |
|  |  |  |  |  |  | **1×** | **20×** |
| LA1924 | 25.46 | 99.13 | 95.49 | 36.55 | 99.56 | 95.41 | 79.55 |
| MM | 21.64 | 99.26 | 95.81 | 35.96 | 99.01 | 99.45 | 76.63 |
| HSSC | 26.32 | 99.09 | 95.30 | 36.11 | 99.60 | 99.71 | 85.46 |
| LSSC | 31.89 | 99.24 | 96.07 | 35.62 | 99.40 | 99.75 | 93.17 |

HSSC: high-SSC bulked pool; LSSC: low-SSC bulked pool.

**Table S2** **List of InDel primers used for QTL mapping.**

| **Marker** | **Position** | **Forward primer (5' -> 3')** | **Reverse primer (5' -> 3')** |
| --- | --- | --- | --- |
| 2-B1 | SL4.0ch02:2116985 | TGCCTCCTTCACCTTTGGTA | ATCATTCCCCGTTGAGTCTG |
| 2-B2 | SL4.0ch02:5436027 | GGCTCGGAACCTAAAGAAGTG | GAAACCTTGAGAATTGTACTTTGTGA |
| 2-B3 | SL4.0ch02:12040604 | TGTCTAATAGCTTAAAAGACCTCTGA | TGAATAACGCCGATGAACAA |
| 2-B4 | SL4.0ch02:16614909 | GGACAAATAGGAATTCTAAATGGA | AGCTAAATACTGATGTGGGAACA |
| 2-B5 | SL4.0ch02:21711309 | TGGCAATTACAATTTTTCCCTT | AAATTCCACTATTGAGTTTAGAGAACA |
| 2-B6 | SL4.0ch02:25656695 | TTCCACAGTTCTAATTGGCTG | TGTCTTGCAAGTATTTCAGAATCC |
| 2-B7 | SL4.0ch02:31581976 | GCTCAACTAATTGAAGCAGATCTAAA | CATGGAGTTGTCAATTTGTTTTG |
| 2-B8 | SL4.0ch02:37135407 | TTTGATAGTCAACATAACCCAAGTT | TGTGGCCATCAGGCATATTA |
| 2-B9 | SL4.0ch02:37255083 | TTCGAAGCACCAAACCAAAT | TGGCTTTTGTTTCTTACTGAGTTG |
| 2-B10 | SL4.0ch02:41684079 | GCTTTCGCGGCTATCAAGC | TTTCACCGCGCACAACTG |
| 2-B11 | SL4.0ch02:42109166 | AGCTCGCTTTAGTTGCG | TGGCTGGGCTATTTGTTTC |
| 2-B12 | SL4.0ch02:46638665 | CAAGTGTCTTGGGAACTTTGC | TGAGTCTCAACACGTTATCAAACAT |
| 2-B13 | SL4.0ch02:49106548 | GCACCTACATTCAAAACATTGC | ACCACATGCACACAAGGAAG |
| 2-B14 | SL4.0ch02:53014114 | GCTTGAGATGCGAATGAGAA | CAGGCAGCAGATGTATGCAA |
| 7-B1 | SL4.0ch07:275854 | CATGCAAAGTGAGGCCAAC | TATCGGCCAGAAGCAGATAC |
| 7-B2 | SL4.0ch07:280054 | GCTCGAACCCGAACTTCTTG | CGTATTCGCGTAGGTCCTC |
| 7-B3 | SL4.0ch07:907591 | GACGCAGAGAGTAGGTACGA | CTCTTGCCTAAGCTTCGTG |
| 7-B4 | SL4.0ch07:1203128 | GAGGAGCTAACGATTTGCTG | ATGCTATGCAAACCGCTAG |
| 7-B5 | SL4.0ch07:1521426 | CGGATCTGACGCCTAGTAG | TTGGGCCAATTGCTGC |
| 7-B6 | SL4.0ch07:1665410 | GTTGCCCCTTTCAGCTATG | GGAATTGGAAGGGGCTTGA |
| 7-B7 | SL4.0ch07:1683272 | CAAACCAAAATAGAGAAATCAACA | TAAAATGCATGCCCTTGACG |
| 7-B8 | SL4.0ch07:1754173 | GGTCAAATGAAGTCATCACTCAC | TCTACAGGATCTGCACCAC |
| 7-B9 | SL4.0ch07:1762091 | AAAATTCATTTCATAACTCTTCCCA | TCGGATAATAAGTATCATTCACTTTCA |
| 7-B10 | SL4.0ch07:1860615 | GAGATCCGTTAGGTGGTGGGGA | GAACTTCATTCGGCTTGTTCAGA |
| 7-B11 | SL4.0ch07:2072302 | CCGCTTACAATTGAACCCATC | GACGGGTTATGGCCTTATACG |
| 7-B12 | SL4.0ch07:2209618 | AGTTGTCTCGATATCCATCCG | TACGTAGTTGCAGTGCAGGC |
| 7-B23 | SL4.0ch07:2330621 | TTCTGGTTAAGCCTCTTCCTC | CAACCATACAACTCTTCCGAG |
| 7-B24 | SL4.0ch07:2355645 | GGTTAATACCACTACATTGGTGAAG | GAGTTGGTTGATCGTCTGAC |
| 7-B13 | SL4.0ch07:2386106 | CACACACGTGCACATACACAA | CACTTCGTGAGATCATGCAAA |
| 7-B14 | SL4.0ch07:2419181 | AGCGGTACTCAAAGGATAATATCC | GGACTCATCTCATTTTGTAGTCTG |
| 7-B25 | SL4.0ch07:2466003 | GTCAAGGAATGGAGATACACTCG | GGAAGGTGAGTAGGACCTATTCC |
| 7-B15 | SL4.0ch07:2527112 | TCCAATGCGTACTAGGAGAG | CTCAATGGAGTCGTGTAGAC |
| 7-B26 | SL4.0ch07:2554375 | CGCGTATGCGTATGTGTATG | TGGAATAAAGGTACACTGGCCT |
| 7-B16 | SL4.0ch07:2746157 | AGTGTGTGCACAATCAAACA | AAGACGCGTAGAGAGAAAG |
| 7-B17 | SL4.0ch07:3021346 | TGACCGCAACAACACTTG | GCACTCGCGTTCGAAT |
| 7-B18 | SL4.0ch07:3309779 | GGGAGTTAAGGGTATCACCTC | GCGTAAGAGAACGTGGTTG |
| 7-B19 | SL4.0ch07:3511641 | GTCTGTAACACGACATTTGAAC | ATGACGATGAACTGTTGCC |
| 7-B20 | SL4.0ch07:3750618 | AAGTATCACGTGAACTAGCCA | AACTTGAGTGAACGCTACTCA |
| 7-B21 | SL4.0ch07:5694359 | TTCCAAAAATTGTCCCAACC | CGGACGAAGAAACCACAAAT |
| 7-B22 | SL4.0ch07:5803657 | TTTGAAATAGTCATAACGAGAACAAC | AGACCCTTTTCCCTTCCCTT |
| 9-B1 | SL4.0ch09:99492 | GATTAGTCCAGCTTCCGGTG | CGCGTCCCTTCTACCATTT |
| 9-B2 | SL4.0ch09:3382292 | GGTGATGTAGGTCGTTTAGTC | TTTCTCGGGGGGAAAAAAC |
| 9-B3 | SL4.0ch09:3523311 | CTCCACACTAGACGGAACC | GCCTTTCTTCCAGGATGC |
| 9-B4 | SL4.0ch09:6864845 | AGCATGCACGTCAATACTGG | TCCTCCTAAGTTGTGTATCTTCATTG |
| 9-B5 | SL4.0ch09:13915611 | TCCAACATGTACCATGAGAAGC | CAACGTTTTAATCTTGACCCA |
| 9-B6 | SL4.0ch09:19325481 | AATTGTTGATGCAATGCTGA | TGCATTCCGATTAAAGGAAAA |
| 9-B7 | SL4.0ch09:25785599 | AAATGGATAAGAACGCGGTG | CTGCATCCATGGAAGAACAA |
| 9-B8 | SL4.0ch09:35016494 | TTGATTCATTGTAGAAATAGGGC | GCTCCCTCTTCCAGGCTTAG |
| 9-B9 | SL4.0ch09:39291818 | TGCACATTTTTCTGCTGAGTG | TGATTTAGTGTGCCTTTTTATGC |
| 9-B10 | SL4.0ch09:46626854 | GATGAACATAATATGCATTGACCTC | TTGGGTGCCTTACCATTTTT |
| 9-B11 | SL4.0ch09:56697059 | GAGAGGAAGTACGAGCAGAAGAA | AATTATGGGCACGTTTGGAA |
| 9-B12 | SL4.0ch09:61681607 | TTTCCTACTTGGCGTTCGAC | AAGATGTACTGGGTTGTGCTGA |
| 9-B13 | SL4.0ch09:68375091 | TCTGAGGTTTTTGGGAAACAA | CCTGAAAAAGACCGATCGATAA |

**Table S3** **QTLs for fruit SSC identified in the F_2_ population.**

| **QTL** | **Peak Marker** | **Peak LOD** | **Size (Mb)** | **PVE (%)** | **Add** | **Dom** |
| --- | --- | --- | --- | --- | --- | --- |
| *qSSC2.1* | 2-B11 | 9.19 | 4.95 | 14.91 | -0.79 | 0.18 |
| *qSSC7.1* | 7-B14 | 4.14 | 0.32 | 6.50 | -0.41 | 0.12 |
| *qSSC9.1* | 9-B3 | 12.22 | 10.53 | 17.78 | -0.65 | 0.51 |

**Table S4 List of primers used for *STP1* and *LIN5* amplify.**

| **Primers** | **Sequence (5' -> 3')** | **Gene** |
| --- | --- | --- |
| 2JC-F | F: CCTTGCGGTTGAAGTAAACG | *STP1* |
| 2JC-R | R: GCATTTGGTCGCACATGA |  |
| 9JC-F | F: CTACAGCTTGGATGGGCCAAGATG | *LIN5* |
| 9JC-R | R: CAAATTGTTCGGCCTCGTTCAAAC |  |

**Table S5 Annotations of 13 candidate genes for *qSSC7.1*.**

| **Gene ID** | **Position** | **Functional Prediction** |
| --- | --- | --- |
| Solyc07g007710 | SL4.0ch07:2388634..2397955 (-) | Defensin protein |
| Solyc07g007720 | SL4.0ch07:2343192..2343834(-) | Unknown Protein |
| Solyc07g007730 | SL4.0ch07:2347167..2348735 (-) | Defensin protein |
| Solyc07g007735 | SL4.0ch07:2351154..2352944 (-) | Defensin protein |
| Solyc07g007740 | SL4.0ch07:2354988..2355403 (-) | Defensin protein |
| Solyc07g007750 | SL4.0ch07:2361999..2363288 (-) | Defensin protein |
| Solyc07g007755 | SL4.0ch07:2366352..2367380 (-) | Defensin protein |
| Solyc07g007770 | SL4.0ch07:2378430..2379136 (+) | Unknown Protein |
| Solyc07g007780 | SL4.0ch07:2388634..2397955 (-) | Calmodulin-binding receptor-like cytoplasmic kinase 3 |
| Solyc07g007790 | SL4.0ch07:2410744..2419566 (+) | **Sucrose-phosphate synthase** |
| Solyc07g007800 | SL4.0ch07:2426248..2429334 (+) | Heptahelical transmembrane protein 1 |
| Solyc07g007810 | SL4.0ch07:2430514..2447866 (+) | N-alpha-acetyltransferase 35 |
| Solyc07g007820 | SL4.0ch07:2453950..2464794 (+) | ENTH/ANTH/VHS superfamily protein |

**Table S6** **Sequence variations in the 2.7 kb upstream and coding region of *SlSPS1*.**

| **Position** | **Distance to start codon (bp)** | **Variation type** | **MM sequence** | **LA1924 sequence** |
| --- | --- | --- | --- | --- |
| SL4.0ch07:2408575 | -2355 | InDel | A | AG |
| SL4.0ch07:2408758 | -2172 | SNP | C | T |
| SL4.0ch07:2408877 | -2053 | SNP | T | C |
| SL4.0ch07:2408896 | -2034 | SNP | C | T |
| SL4.0ch07:2409079 | -1851 | SNP | T | C |
| SL4.0ch07:2409349 | -1581 | InDel | T | TT |
| SL4.0ch07:2409450 | -1480 | SNP | T | G |
| SL4.0ch07:2409557 | -1373 | SNP | A | G |
| SL4.0ch07:2410166 | -764 | SNP | A | G |
| SL4.0ch07:2410172 | -758 | SNP | T | C |
| SL4.0ch07:2410342 | -588 | SNP | G | A |
| SL4.0ch07:2410379 | -551 | SNP | G | A |
| SL4.0ch07:2410416 | -514 | SNP | G | C |
| SL4.0ch07:2410718 | -213 | InDel | TT | T |
| SL4.0ch07:2410816 | -114 | InDel | TT | T |
| SL4.0ch07:2411015 | +86 | SNP | A | G |
| SL4.0ch07:2417473 | +6544 | SNP | G | T |

**Table S7** **List of primers used for RT-qPCR, [functional verification](https://www.iciba.com/word?w=functional%20verification), and reporter assays.**

| **Primer name** | **Sequence (5' -> 3')** | **Purpose** |
| --- | --- | --- |
| ACTIN-F | GGGATGGAGAAGTTTGGTGGTGG | RT-qPCR |
| ACTIN-R | CTTCGACCAAGGGATGGTGTAGC |  |
| qSlSPS1-F | CTGGTGGTCAGGTGAAGTATGTT |  |
| qSlSPS1-R | TCTAGTAAGCAAGTCAACCCGATA |  |
| OESlSPS1-F | acgggggactcttgaggatccATGGCGGGAAACGATTGG | Over expression |
| OESlSPS1-R | agatccccgggtaccgagctcTCCTTTGAGTACCGCTAGTTTCTCC |  |
| CRSlSPS1-F | TTCATCTCGGGTCTCGTTTGTGAGGGAGAGCAAGCTCGGGTTTTAGAGCTAGAAATAG | CRISPR/Cas9 |
| CRSlSPS1-R | TTCATCTCGGGTCTCTAAACGAGGCCGATTGCCTAGAATTGACCGTTGATAGTGGATAG |  |
| 25GUSF | tgggcccggcgcgccaagcttCGTGTTTCACTCGCGTATAAATG | GUS reporter assays |
| 20GUSF | tgggcccggcgcgccaagcttCAGCTTTATGCACAGTCTCTCGG |  |
| 15GUSF | tgggcccggcgcgccaagcttGATGAATTTAGTTCCATTCAAATGCA |  |
| 7GUSF | tgggcccggcgcgccaagcttGAATGTATCCGAACCATCATCGT |  |
| GUSR | ggtggactcctcttagaattcCTCCTCCGCCGCACTCCT |  |
| 25LUCF | ggtaccgggccccccctcgagCGTGTTTCACTCGCGTATAAATG | LUC reporter assay |
| 20LUCF | ggtaccgggccccccctcgagCAGCTTTATGCACAGTCTCTCGG |  |
| LUCR | ggcggccgctctagaactagtCTCCTCCGCCGCACTCCT |  |
| L1-LUCF | ggtaccgggccccccctcgagTTTATTCCATAATTAGAAATGAAACTATCC |  |
| L1-LUCR | ggcggccgctctagaactagtGTTGGTTCTAGATTTTTGAGGAATAAAA |  |
| L2-LUCF | ggtaccgggccccccctcgagTGCGTTGAAAGGACCCACA |  |
| L2-LUCR | ggcggccgctctagaactagtCCGCTCCAAATCCCGTGG |  |

**Supplementary Materials and Methods**

**Plant materials and population Construction**

For genetic analysis of soluble solids content (SSC), a cross was made using the high‑SSC wild tomato accession *S. pimpinellifolium* LA1924 as the paternal parent and the low‑SSC cultivated tomato *S. lycopersicum* ‘Moneymaker’ (MM) as the maternal parent. The SSC of LA1924 was consistently and relatively high when evaluated under diverse environmental conditions across multiple trials: 12.5% in an open field in Shunyi, Beijing (July 2014); 12.8%–16.5% in Korla, Xinjiang (August 2015); and 11.4% in a greenhouse in Changping, Beijing (November 2016). An initial F₂ population of 300 individuals was developed in spring 2023 for QTL mapping, and a larger F₂ population consisting of 2,100 plants was further generated for fine-mapping. All plant materials were grown in glasshouse at Changping Nankou Station of the Chinese Academy of Agricultural Sciences under standardized irrigation, fertilization, and environmental control conditions.

**Phenotypic investigation and data analysis**

For SSC determination, fruit sampling and measurement were conducted three times per individual plant. Fruit SSC was measured using a handheld refractometer (PAL-1, ATAGO Co., Ltd., Tokyo, Japan). Phenotypic data were recorded in Microsoft Excel 2016 and statistically analyzed using GraphPad Prism 10. Differences between groups were assessed using Student’s *t*-test, with *p* < 0.05 considered statistically significant. For comparisons involving three or more groups, one-way ANOVA was conducted using the aov() function, and post-hoc multiple comparisons were performed using the TukeyHSD() function. All statistical analyses were performed in *R* (version 4.5.1). Figures were generated using GraphPad Prism 10 and assembled in Adobe Illustrator.

**Bulk Segregant Analysis Sequencing (BSA-seq)**

From the F₂ segregating population (n=300), two extreme-phenotype bulks were constructed: a high-SSC bulk consisting of the 20 individuals with the highest SSC values, and a low-SSC bulk consisting of the 20 individuals with the lowest SSC values. Paired with the parental lines LA1924 and MM, the four samples were subjected to whole-genome sequencing on the Illumina platform, yielding 150-bp paired-end reads with ~20× mean coverage. Raw reads were cleaned using fastp v0.23.2 (Chen et al., 2018) to remove adapters, trim poly-G tails and low-quality bases. Clean reads were aligned to the *S. lycopersicum* reference genome (Heinz1706 SL4.0) via BWA-MEM v0.7.17 (Li & Durbin 2009). Alignments were converted to BAM format and sorted with SAMtools v1.12 (Li et al. 2009). SSC-associated genomic regions were identified using the ΔSNP-index method (Takagi et al. 2013). Single nucleotide polymorphisms (SNPs) indices were calculated in 100 kb sliding windows with 10-kb steps; genomic intervals exceeding the 95^th^ and 99^th^ percentile confidence threshold (1,000 permutations) were designated candidate QTL regions.

**Molecular marker development and genotyping**

Based on BSA-seq results, polymorphic insertions/deletions (InDels) loci were identified from parental resequencing data within the candidate QTL intervals significantly associated with tomato fruit SSC, namely *qSSC2.1*, *qSSC7.1*, and *qSSC9.1*. Specific primers were designed and synthesized for these polymorphic InDel markers (Table S2), Genotyping was performed by PCR amplification using genomic DNA as the template, followed by agarose gel electrophoresis to distinguish different alleles based on the size differences of PCR products.

To verify sequence variation at the known functional loci underling *qSSC2.1* and *qSSC9.1*, locus-specific primers flanking were designed (Table S4). The resulting PCR amplicons were purified and analyzed by Sanger sequencing to confirm parental polymorphisms.

**Linkage map construction and fine mapping**

The polymorphic InDel markers within the candidate QTL intervals were used for genotyping the F_2_ population, and a genetic linkage map was constructed using QTL IciMapping software. QTL mapping was subsequently performed by integrating genotypic data with SSC phenotypes, with a LOD score threshold of ≥ 2.5 set to declare the preliminary interval of the target QTL. For fine-mapping, individuals heterozygous across the initial QTL interval were identified from the expanded F₂ population (n=2,100), and self-pollinated to produce F₂:₃ families, which were used to narrow the QTL interval. Within the refined interval, heterozygous recombinants were then chosen to generate F₃:₄ progenies. For each recombinant family, 12 plants homozygous for either parental allele (LA1924/MM) were selected and phenotyped. Through iterative marker development and recombinant screening, the candidate interval was gradually refined.

**Vector construction and plant transformation**

Two sgRNA target sites within the coding sequence of *SlSPS1* were predicted using the online CRISPR-GE toolkit ([http://skl.scau.edu.cn/](http://skl.scau.edu.cn/)). Both sites were located in the 5' coding region, preferentially within the first exon, and were selected for their high target specificity and low off-target potential. The corresponding primers were synthesized (Table S7) and used to amplify the sgRNA expression cassette by PCR with the pCBC-S1 plasmid as template. The PCR amplicons and pMGET backbone were digested with *BsaI*, and the sgRNA cassette was ligated into the linearized vector using T4 DNA ligase to generate the CRISPR/Cas9 editing construct (Xing et al., 2014).

For the overexpression construct, the full-length coding sequence of *SlSPS1* was amplified from LA1924 cDNA and inserted into the *BamHI/SacI*-digested pCAMBIA1300 backbone via homologous recombination to generate 35S::*SlSPS1*-GFP construct. All recombinant plasmids were transformed into *Agrobacterium tumefaciens* strain GV3101 for tomato transformation (Sharma et al., 2009). The MM was used as the recipient for overexpression, the LA1924 and M82 were used for CRISPR/Cas9-mediated gene editing. Transgenic overexpression lines were validated by T-DNA insertion detection, and gene-edited lines were genotyped to identify mutation types at the target sites. T_1_ transgenic plants were used for SSC determination, and total RNA was isolated for *SlSPS1* expression analysis.

**RNA extraction and reverse transcription quantitative PCR (RT-qPCR) analysis**

Total RNA was extracted from tomato leaf and fruit tissues using a plant Quick RNA extraction kit (Huayueyang Biotech, Beijing, China; 0416-50gk). First-strand cDNA was synthesized using a reverse transcription kit (YEASEN, Shanghai, China; 11141ES60). RT-qPCR was performed on a Roche LightCycler^®^ 480 system using Hieff UNICON^®^ Universal Blue qPCR SYBR Master Mix (YEASEN). *ACTIN* (*Solyc11g005330*) served as reference genes for normalization. All primers were designed with Primer Premier 5.0 software (see Table S7 for sequences).

**Promoter activity analysis**

The ~2.7-kb regions upstream of the ATG start codon of *SlSPS1* were amplified from genomic DNA of the two parental lines, LA1924 and MM. These full-length promoters were truncated by PCR into four fragments spanning: S1: −2,588 to −1 bp; S2: −2,106 to −1 bp; S3: −1,546 to −1 bp; S4: −697 to −1 bp, respectively (Xu et al., 2014). Each fragment was cloned into the pCAMBIA1391-GUS vector in replace the native 35S promoter to drive the GUS reporter gene, yielding promoter–GUS fusion constructs. For dual-luciferase assays, the S1 and S2 fragments from both parental lines were inserted into the pGreenII 0800-LUC vector to generate firefly luciferase (LUC) reporter constructs. In addition, the 2106-to-2588-bp interval of each promoter, which contained the polymorphic sites, was divided into two subfragments (L1: −2,500 to −2,265 bp, and L2: −2,254 to −2,107 bp), each harboring one variant; these subfragments were also cloned into the pGreenII 0800-LUC. All constructs were verified by sequencing. The plasmids were co-transformed with the helper plasmid the pSoup-p19 into *Agrobacterium tumefaciens* strain GV3101.

*Nicotiana benthamiana* plants were grown at 28°C under a 16 h light/8 h dark photoperiod until reaching the six- to eight-leaf stage, then used for *Agrobacterium-mediated* transient infiltration. Prior to infiltration, *Agrobacterium* cells were resuspended in 10 mM MES buffer (pH 5.7) supplemented with 10 mM MgCl₂, adjusted to a final OD600 of 1.0, and subjected to static incubation in the dark at 28°C for 2–3 h (Wu et al., 2014).

GUS activity of each sample was measured using the GUS Gene Quantitative Detection Kit (Kulaibo, SL7161) following the manufacturer's instructions strictly. Luciferase activity was measured using a Dual-Luciferase Reporter Assay System (Promega, E1910) according to the manufacturer’s instructions, and relative transcriptional activity was expressed as the LUC/REN ratio.

**Histochemical GUS staining**

Agroinfiltrated *Nicotiana benthamiana* leaves were fixed and partially cleared in 80% acetone on ice for 20–30 min, washed 2–3 times with sterile water, and then immersed in freshly prepared GUS staining solution. Vacuum infiltration was performed for 2 min per round for 2–3 rounds until the leaves were fully infiltrated, followed by overnight incubation at 37°C in the dark. After staining, leaves were destained with 80% ethanol until the negative control was fully bleached.

**Allele frequency analysis**

Resequencing data used for allele frequency analysis of tomato populations were obtained from the NCBI database under the following BioProjects: PRJNA259308, PRJNA353161, PRJNA454805, PRJEB5235, PRJNA557253, PRJEB5226, PRJEB5227, and PRJEB5228. Resequencing data for certain hybrid accessions were not included because the corresponding studies are still ongoing and the data have not yet been released. Variant calling was performed as previously described (Li et al., 2024). Allele frequencies of the SlSPS1 promoter InDel were analyzed in four tomato groups: S. pimpinellifolium (PIM), S. lycopersicum var. cerasiforme (SLC), cultivated S. lycopersicum (SLL), and F_1_ hybrids.

**References**

Chen, S., Zhou, Y., Chen, Y. and Gu, J. (2018) fastp: an ultra-fast all-in-one FASTQ preprocessor. *Bioinformatics* 34, i884–i890.

Hu, W., Chen, Y., Xu, Z., Liu, L., Yan, D., Liu, M., Yan, Q., Zhang, Y., Yang, L., Gao, C., Liu, R., Qin, W., Miao, P., Ma, M., Wang, P., Gao, B., Li, F. and Yang, Z. (2024) Natural variations in the Cis-elements of *GhRPRS1* contributing to petal colour diversity in cotton. *Plant Biotechnol. J*, 22, 3473-3488.

Li, H., Handsaker, B., Wysoker, A., Fennell, T., Ruan, J., Homer, N., Marth, G., Abecasis, G., Durbin, R. and 1000 Genome Project Data Processing Subgroup (2009) The Sequence Alignment/Map format and SAMtools. Bioinformatics 25, 2078–2079.

Li, H., & Durbin, R. (2009) Fast and accurate short read alignment with Burrows–Wheeler transform. *Bioinformatics*, 25, 1754–1760.

Li, X., Liu, X., Pan, F., Hu, J., Han, Y., Bi, R., Zhang, C., Liu, Y., Wang, Y., Liang, Z., Zhu, C., Guo, Y., Huang, Z., Wang, X., Du, Y., Liu, L. and Li, J. (2024) Dissection of major QTLs and candidate genes for seedling stage salt/drought tolerance in tomato. BMC Genomics, 25, 1170.

McKenna, A., Hanna, M., Banks, E., Sivachenko, A., Cibulskis, K., Kernytsky, A., Garimella, K., Altshuler, D., Gabriel, S., Daly, M. and DePristo, M.A. (2010) The Genome Analysis Toolkit: a MapReduce framework for analyzing next-generation DNA sequencing data. Genome Res. 20, 1297–1303.

Sharma, M.K., Solanke, A.U., Jani, D., Singh, Y. and Sharma, A.K. (2009) A simple and efficient Agrobacterium-mediated procedure for transformation of tomato. *J Biosci.* 34, 423–433 .

Takagi, H., Abe, A., Yoshida, K., Kosugi, S., Natsume, S., Mitsuoka, C., Uemura, A., Utsushi, H., Tamiru, M., Takuno, S., Innan, H., Cano, L.M., Kamoun, S. and Terauchi, R. (2013) QTL-seq: rapid mapping of quantitative trait loci in rice by whole-genome resequencing of DNA from two bulked populations. Plant J. 74, 174–183.

Wang, K., Li, M. and Hakonarson, H. (2010) ANNOVAR: functional annotation of genetic variants from high-throughput sequencing data. Nucleic Acids Res. 38, e164.

Waese J, Fan J, Pasha A, Yu H, Fucile G,Shi R,et al. (2017) ePlant: Visualizing and Exploring Multiple Levels of Data for Hypothesis Generation in Plant Biology. *The Plant Cell.* 29, 1806–1821.

Xing, H.L., Dong, L., Wang, Z.P., Zhang, H.Y., Han, C.Y., Liu, B., Wang, X.C. and Chen, Q.J. (2014) A CRISPR/Cas9 toolkit for multiplex genome editing in plants. *BMC Plant Biol.* 14, 327 .

Xu, Y.Z., Kanagaratham, C., Jancik, S., Radzioch, D. (2013) Promoter Deletion Analysis Using a Dual-Luciferase Reporter System. In: Bina, M. (eds) Gene Regulation. *Methods in Molecular Biology*, 977, 79-91.
